# Expression of Wild-Type CFTR Suppresses NF-κB-Driven Inflammatory Signalling

**DOI:** 10.1371/journal.pone.0011598

**Published:** 2010-07-14

**Authors:** Mairi J. Hunter, Kate J. Treharne, Alexandra K. Winter, Diane M. Cassidy, Stephen Land, Anil Mehta

**Affiliations:** Division of Medical Sciences, Ninewells Hospital and Medical School, University of Dundee, Dundee, United Kingdom; Ludwig-Maximilians-Universität München, Germany

## Abstract

**Background:**

Mutation of the cystic fibrosis transmembrane-conductance regulator (CFTR) causes cystic fibrosis (CF) but not all CF aspects can easily be explained by deficient ion transport. CF-inflammation provides one example but its pathogenesis remains controversial. Here, we tested the simple but fundamental hypothesis that wild-type CFTR is needed to suppress NF-κB activity.

**Methodology/Principal Findings:**

In lung epithelial (H441) and engineered (H57) cell lines; we report that inflammatory markers are significantly suppressed by wild-type CFTR. Transient-transfection of wild-type CFTR into CFTR-naïve H441 cells, dose-dependently down-regulates both basal and Tumour Necrosis Factor-α evoked NF-κB activity when compared to transfection with empty vector alone (p<0.01, n>5). This effect was also observed in CFTR-naïve H57-HeLa cells which stably express a reporter of NF-κB activity, confirming that the CFTR-mediated repression of inflammation was not due to variable reporter gene transfection efficiency. In contrast, H57 cells transfected with a control cyano-fluorescent protein show a significantly elevated basal level of NF-κB activity above control. Initial cell seeding density may be a critical factor in mediating the suppressive effects of CFTR on inflammation as only at a certain density (1×10^5^ cells/well) did we observe the reduction in NF-κB activity. CFTR channel activity may be necessary for this suppression because the CFTR specific inhibitor CFTR_inh172_ significantly stimulates NF-κB activity by ∼30% in CFTR expressing 16HBE14o− cells whereas pharmacological elevation of cyclic-AMP depresses activity by ∼25% below baseline.

**Conclusions/Significance:**

These data indicate that CFTR has inherent anti-inflammatory properties. We propose that the hyper-inflammation found in CF may arise as a consequence of disrupted repression of NF-κB signalling which is normally mediated by CFTR. Our data therefore concur with in vivo and in vitro data from Vij and colleagues which highlights CFTR as a suppressor of basal inflammation acting through NF-κB, a central hub in inflammatory signalling.

## Introduction

Cystic fibrosis (CF) is widely regarded solely as a defect in ion transport arising from a heritable mutation in the cystic fibrosis transmembrane conductance regulator (CFTR) [1]. CFTR was identified nearly 20 years ago [2], [3], [4] but it remains controversial as to why defects in this ion channel can cause persistent pulmonary inflammation given that inflammation may occur in the absence of recognised CF pathogens [5], [6]. Whatever the mechanism(s), sustained inflammation of the CF epithelium is the cause of most morbidity and premature mortality in CF patients. Numerous studies report that signalling through the nuclear factor κB (NF-κB) pathway, a central hub in inflammatory signalling, is increased in CF lungs and that this results in excess pro-inflammatory cytokines such as interleukin 8 (IL-8) [7], [8]. One school of thought believes that this excess is provoked by underlying infection(s) in the lungs. However, recent data suggest that an excess of these inflammatory markers may even be present in the sterile uterine environment of CF fetuses prior to any direct exposure to pathogens. For example, Verhaeghe *et al.* showed that there is increased activation of a number of NF-κB driven genes in a 24 week old CF fetus compared to non-CF fetuses of similar age (22 weeks and 23 weeks) [9], whilst others report that in CF and non-CF human fetal tracheal grafts explanted under the skin of immuno-deficient ‘SCID’ mice, there was a significantly increased intraluminal IL-8 content and consistently elevated accumulation of murine leukocytes in the sub-epithelial region in the CF grafts compared to controls [10]. These data are consistent with reports showing increased activation of NF-κB in a variety of CF cell lines [11], [12] where infection is not an issue. In agreement, some studies have described the presence of neutrophils and elevated levels of IL-8, in the absence of any detected pulmonary pathogens in bronchoalveolar lavage fluids of CF newborns as compared to healthy individuals [13], [14]. The combined body of evidence suggests that the chronic inflammation observed in CF is either due to some action of the mutant CFTR gene product itself (a knock-in mutant function) or is invoked by the absence of some normal function of the wild type protein (or both may apply). In this paper we focus on the effects of the wild type protein on the inflammatory markers NF-κB and IL-8.

Recently Vij *et.al.* proposed a new role for wild type CFTR towards inflammation, suggesting that introduction of CFTR to a previously naïve cell line was sufficient to significantly reduce basal IL-8 secretion [15]. Here, we test this idea by examining the inflammatory consequences of a cell ‘seeing’ wild type CFTR cDNA either for the first time in transiently transfected CFTR-naïve cells or in the context of elevating pre-existing CFTR levels by transient over-expression above endogenous CFTR baseline concentrations.

## Results

### Wild type CFTR specifically suppresses inflammation in a number of cell lines

**Figure 1:**
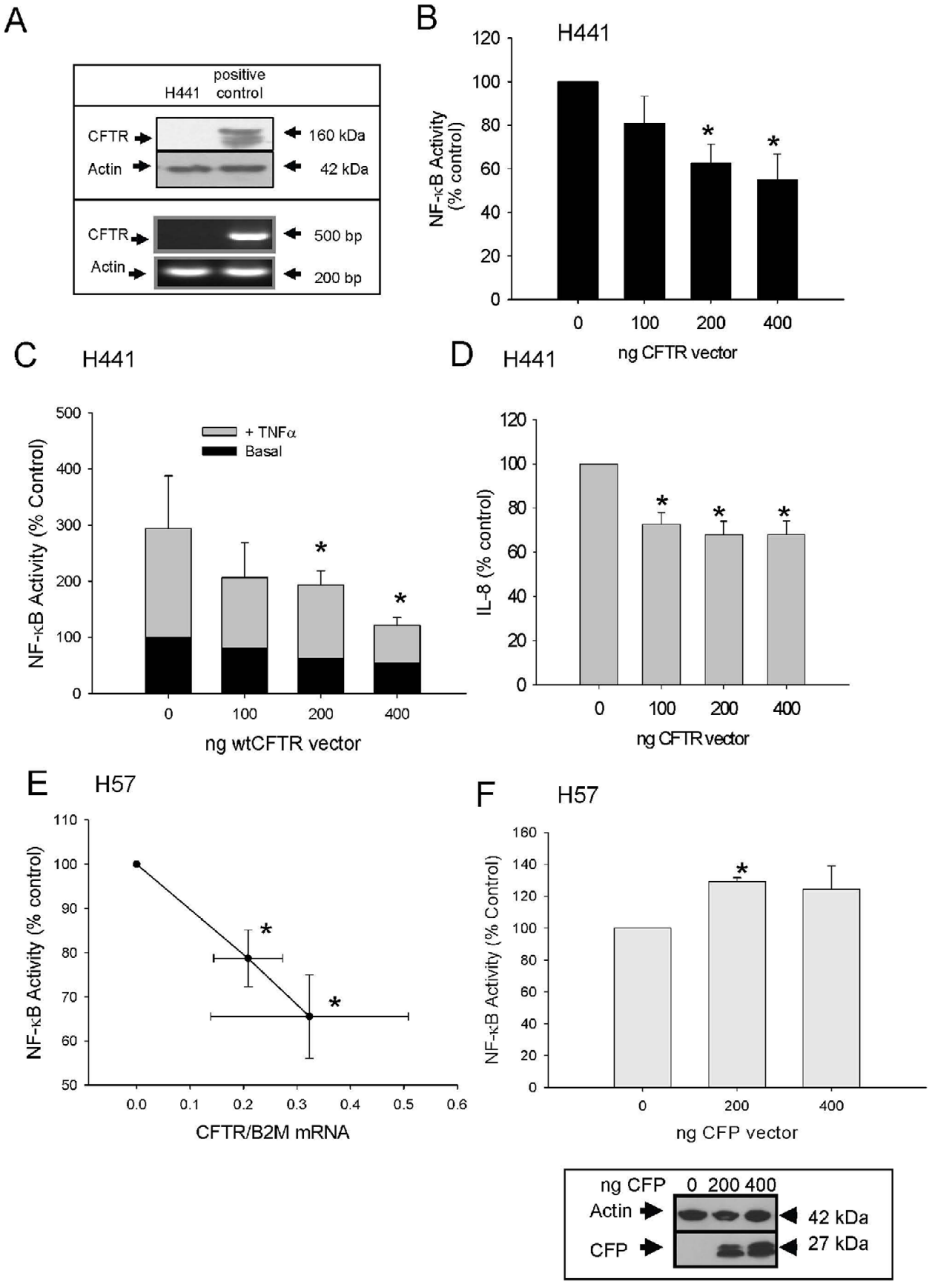
Wild-type CFTR attenuates both basal and induced inflammation. **A**. H441 cells do not express CFTR protein as shown by western blot or CFTR mRNA as shown by RT-PCR (representative of 3 experiments). **B**. NF-κB activity in H441 cells transfected with increasing amounts of wt CFTR cDNA (0–400 ng, n = 5, *p<0.01). **C**. NF-κB activity in H441 cells transfected with increasing amounts of wt CFTR and treated with 10ng TNFα for 4 hours (open bars, n = 5, * p<0.001). Closed bars represent basal levels of activity re-drawn from figure 1B. **D**. The levels of IL-8 in the supernatants from B were measured by the ELISA method, n = 3, * p<0.01. **E** NF-κB activity (y-axis) in H57 cells transfected with increasing amounts of wt CFTR cDNA (0–400 ng), n = 5, *p<0.05. The amount of CFTR mRNA was then analysed by real-time PCR (qPCR) (x axis). There was no CFTR mRNA detected in the H57 cells prior to transfection, thus these treatments were allocated the nominal value of zero in all cases. **F** NF-κB activity in HeLa H57 cells transfected with increasing amounts of CFP vector (0–400 ng) n = 4, *p<0.05. Inset shows CFP levels following transfection with CFP vector.

To test the proposal that wild-type CFTR (wt-CFTR) could itself suppress inflammation, a wt-CFTR expressing vector was transfected into the pulmonary epithelial cell line, H411 as detailed in the methods to measure its effects on NF-κB activity. Our H441 clones did not express CFTR as shown by both Western blot and RT-PCR (figure 1A). The NF-κB activity observed with empty vector transfection during co-transfection with a NF-κB driven reporter construct was significantly reduced by the presence of wt-CFTR with wt-CFTR acting in a dose-dependent manner resulting in a ∼45% reduction in NF-κB activity after the addition of 400 ng of wt-CFTR vector (p<0.05, figures 1B, C). Most of the reduction occurred by transfecting as little as 200 ng of wt-CFTR-encoding plasmid. This significant reduction in NF-κB activity was mirrored by a significant decrease in IL-8 in the supernatant (figure 1D) indicating that for this lung cell line, the cell signalling path from NF-κB to the NF-κB-response element in the IL-8 gene was wt-CFTR sensitive. Next, we further explored the idea proposed by Vij *et al.* whereby active CFTR contained within lipid rafts at the cell membrane associates with a TNFα-IL1β-TLR receptor complex to tonically repress signalling to the NF-κB pathway. Parallel batches of H441 cells were treated simultaneously with 10 ng/ml TNFα to further stimulate inflammation. TNFα induced a three fold elevation in NF-κB activity in the presence of empty vector (figure 1C, compare heights of first open versus closed bars). Transient wt-CFTR transfection once again retarded the TNFα-induced elevation of NF-κB activity, again acting in a dose dependent, suppressive manner (figure 1C; at 400 ng DNA, TNFα-mediated elevation of NF-κB activity returned to ‘baseline’). Note that ‘baseline’ now refers to empty vector baseline and not the depressed levels of NF-κB activity found in figure 1B (compare heights of last open and closed bars in figure 1C; closed bar data from figure 1B).

Next, H57 cells were tested because they stably express a NF-κB driven luciferase construct thus eliminating any potential co-transfection efficiency issues for the luciferase-based NF-κB reporter assays reported above. Quantitative real-time PCR was used to show that these cells also expressed no detectable endogenous CFTR and that the input of wt-CFTR cDNA once again produced a dose-dependent increase in CFTR transgene expression (figure 1E and data not shown). Once again, we observed a significant ∼35% decrease in base line NF-κB-driven luciferase activity when transfected with 400 ng of a CFTR-expressing plasmid relative to empty vector alone (figure 1E; n = 5, p<0.05).

To further prove that suppression was a CFTR-specific effect, the same experiments were performed using a non-CFTR vector expressing cyano-fluorescent protein (CFP/inset/figure 1F). When the H57 cells were transfected with increasing amounts of CFP expressing vector, we found that there was a significant increase in NF-κB activity of about 20%, further confirming that the significant reduction in NF-κB activity observed in the presence of wt-CFTR, is a CFTR specific phenomenon (figure 1F).

### The CFTR induced reduction in NF-κB Activity is dependent on seeding density

**Figure 2:**
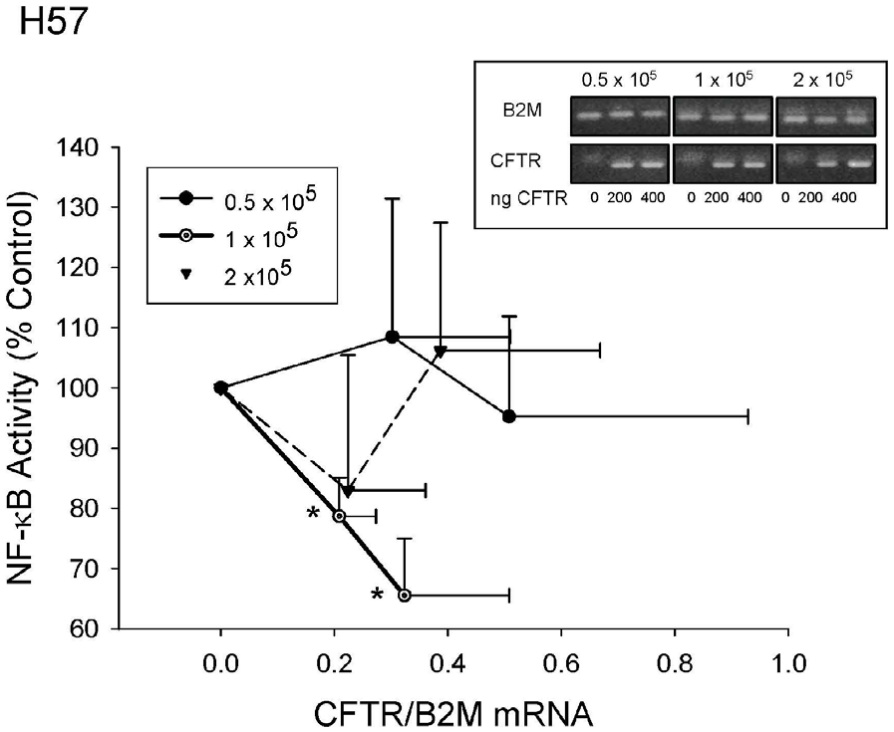
CFTR only reduces inflammation at a critical seeding density. **A**. NF-κB activity (n = 5, *p<0.05, y-axis) in HeLa H57 cells seeded at varying densities (0.5×10^5^, 1×10^5^ and 2×10^5^ cells per well; inset), transfected with increasing amounts of wt CFTR cDNA (0–400 ng), relative to empty vector (100% on y axis). The amount of CFTR mRNA for each transfection as described in methods was then analysed by real-time PCR (qPCR) (x axis and inset). The inset shows final PCR products from a typical qPCR run showing that there was no detectable CFTR mRNA prior to transfection with wt-CFTR vector.

During the course of the investigation, we sometimes found the suppressive effect of CFTR to be much smaller than expected and wondered if this variability was an effect of initial seeding density (starting cell number) relative to NF-κB activity. H57 cells were seeded at 3 different starting densities, each in 12 well plates (0.5×10^5^, 1×10^5^ and 2×10^5^ cells per well) (inset, figure 2). The cells were then transfected 24 h after seeding with increasing amounts of wt-CFTR cDNA supplement to 400 ng with empty vector as above. In parallel, cells were collected for quantitative real-time PCR. Only in cells seeded at 1×10^5^ cells per well (middle panel, figure 2 inset and open circles in main figure) did NF-κB activity decline significantly post CFTR (compare decline with figure 1E). However when expression of CFTR mRNA was determined, this did not differ between wells (data not shown) suggesting that the vector was being expressed at all starting cell numbers (inset, figure 2) but that the cells need to be at certain combination of seeding density and or confluency for the CFTR suppressive effect to occur. Confluency may be a factor because by eye, we noticed that at 2×10^5^ cells per well, where inflammation was not suppressed by CFTR, the cells were almost 100% confluent and thus were presumably contact inhibited, but this could not have been be the sole explanation because the cell plated at 0.5×10^5^ per well were approximately 50% confluent (photomicrographs available on request). This suggests the cells might either require some inter-cell contact or must not be completely confluent for CFTR to significantly reduce a critical marker of inflammation. We interpret this data to suggest that CFTR can but does not always suppress inflammation (see discussion) and further work will have to establish the relationship between proliferation and contact inhibition.

### Pharmacological inhibition of CFTR increases NF-κB activation and IL-8 secretion, while channel activation reduces inflammation

**Figure 3:**
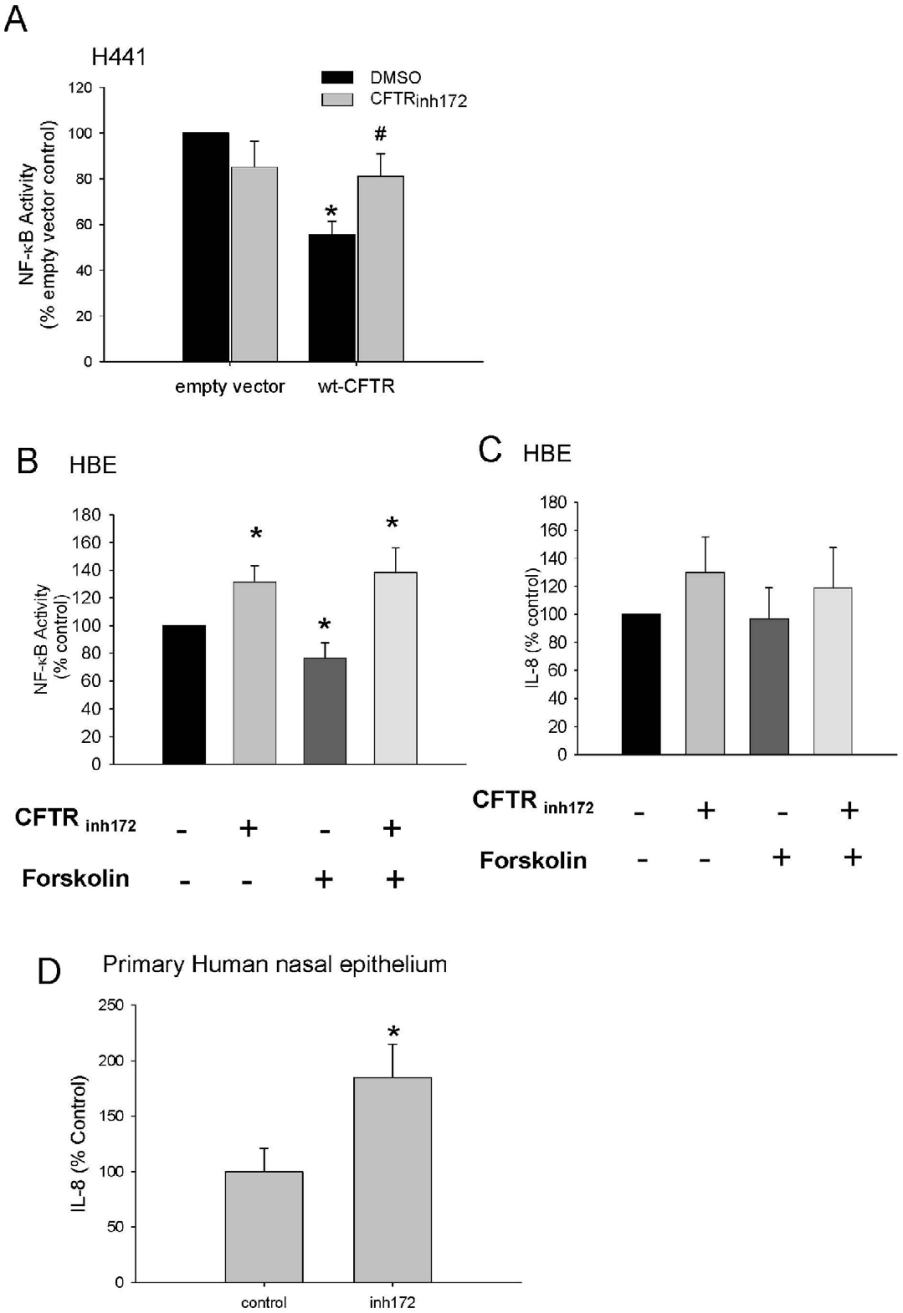
Inhibition of CFTR with CFTR_inh172_. **A**. NF-κB activity in H441 cells transfected with either empty vector or wt-CFTR (400 ng), each treated with 10 µM CFTR_inh172_ for 4 hours, n = 3 *p<0.05 (compared to empty vector control), ^#^p<0.05 (compared to CFTR control). **B**. NF-κB activity in 16HBE14o^−^ cells treated with 10 µM CFTR_inh172_ and/or 10 µM forskolin and 100 µM IBMX for 4 h, n = 7, *p<0.05. **C**. IL-8 levels in supernatants from 16HBE14o^−^ cells measured by ELISA, n = 8, p not significant. **D** IL-8 levels in supernatants from primary human nasal epithelial cells treated with 10 µM CFTR_inh172_ for 4 hours, n = 4, *p<0.05.

Vij *et.al.* proposed that CFTR should be active as an ion channel for the inflammation suppression to occur. CFTR_inh172_ is a relatively specific inhibitor of this chloride channel that may interact with the sixth transmembrane helix in CFTR, probably preventing pore opening [16]. In a recent study it was shown that cells that endogenously express wild-type CFTR mimicked the physiological character of CF cells when treated with this CFTR inhibitor [17]. These data point to the idea that CFTR channel dysfunction can itself cause the expression of pro-inflammatory mediators. Conversely, CFTR channel activity is enhanced, in part, by cAMP-induced activation of PKA, which phosphorylates CFTR thereby opening the channel [18]. To check that this inhibitor was acting in a CFTR specific manner, H441 cells, which do not express CFTR (figure 1), were transfected with either 400ng of empty vector or 400ng wt-CFTR as above. CFTR_inh172_ had no significant effect on basal NF-κB activity in the H441 cells transfected with empty vector (figure 3A, left panels). In contrast, when wt-CFTR was present the suppressive effect of wt-CFTR (closed bar, right panel) was abrogated due to an increase in NF-κB activity induced by the inhibitor (which returned towards the previously elevated baseline, see discussion). To explore the pro-inflammatory effect of this inhibitor on endogenously expressed, rather than transiently transfected CFTR, the normal human CFTR expressing bronchial epithelial cell line 16HBE14o^−^, was used to ascertain whether either pharmacological inhibition or PKA-driven activation of CFTR similarly affects inflammatory markers. 16HBE14o^−^ cells were transiently transfected with the NF-κB luciferase reporter vector as above and 24 h post transfection, they were treated with either the inhibitor (10 µM CFTR_inh172_) and/or the CFTR activator forskolin (10 µM) together with IBMX (100 µM) to sustain elevated cAMP. Following addition of CFTR_inh172_ alone there was a ∼31% increase in NF-κB activation, while treatment with forskolin and IBMX, which is commonly used to open CFTR by raising cAMP [19], resulted in a ∼25% reduction in NF-κB activity (figure 3B, third column). Interestingly, when the cells were simultaneously treated with the inhibitor and the activators of CFTR, NF-κB activity increased by a similar amount to inhibitor alone (figure 3B last column) suggesting dominance of channel-inhibition irrespective of stimuli known to mediate channel-activation (the inhibitor does not alter [cAMP] [20]). Although IL-8 levels in these 16HBE14o^−^ cells were elevated in a similar way to luciferase activity, the trends in these data were not significantly different from one another (figure 3C). This was not pursued further. Instead, we examined the effect of CFTR_inh172_ on a more physiologically relevant model by examining the effect of this CFTR inhibitor on IL-8 secretion in non-CF primary human nasal epithelium cultured *in vitro* in submersion culture (figure 3D). When these cells were treated for 4 h with the inhibitor, there was an 85% increase in IL-8 secretion demonstrating that this CFTR dependent mechanism is also relevant in a primary cell model.

We interpret our data (when considered in the context of the in vivo data from Vij and colleagues) to suggest that pharmacological inhibition of the residual channel function of CFTR, or the equivalent inhibition in the presence of PKA-stimulated channel function, elicit similar pro-inflammatory effects whereas stimuli known to promote CFTR activation may, conversely, suppress inflammation but only provided CFTR is active.

## Discussion

Our studies arose in part from the finding that when CFTR is expressed for the first time in the developing fetal lung, the rapidly rising level of CFTR expression inversely correlated with endogenous NF-κB activity/IL-8 release coupled to our interest in lung development [21]–[25]. Our equivalent data from a lung cancer cell line suggest that functional wild type CFTR can act as a brake on inflammation. To model the scope of this phenomenon under conditions where co-transfection efficiency of our chosen reporter of inflammation was not confounding, we repeated these experiments in a non-lung cell line engineered to stably express a NF-κB driven luciferase vector. Once again the introduction of wt-CFTR into this CFTR-naïve setting significantly reduced endogenous NF-κB activity. This suggests that an anti-inflammatory role of CFTR is a widespread phenomenon with agreement from *in vivo* and in cell data when our findings are compared to those of Vij and colleagues. Remarkably, this agreement occurs despite the fact that all our work was undertaken on undifferentiated sub-confluent cells in submersion culture where we cannot conclude that (open or closed) CFTR was present at an apical membrane or in a particular lipid raft fraction as proposed by Vij and colleagues. Given that CFTR is expressed widely and not just in epithelial cells, our work might have relevance to other cells within the immune system that could play a role in *in vivo* inflammation in CF. Furthermore, in the presence of a relatively specific CFTR inhibitor, we observed an increase in inflammatory markers and conversely, when cAMP was elevated there was a decrease of a similar magnitude below baseline (provided the CFTR inhibitor was absent). The absolute amount of CFTR mediating this effect may be critical to this regulation because the inflammatory markers decreased in a dose-dependent manner with increasing wt-CFTR transfection suggesting that the lack of a threshold level of CFTR is itself an important pro-inflammatory signal. A most interesting finding was that the level of CFTR-induced suppression after TNFα was applied was such that NF-κB-driven luciferase activity only returned to basal levels (found before the stimulus was applied) and never reached the hyper-suppressed nadir observed in the absence of TNFα. These data from figure 1C suggest that CFTR can act both as a suppressor of basal and stimulated inflammation, but in the latter case, CFTR only acts as a post-stimulus, buffer to restrain the magnitude of rise of inflammation following a pro-inflammatory stimulus. This notion is consistent with the frequently observed clinical scenario where CF patients can remain well for prolonged periods but nevertheless succumb rapidly post-infection with a sustained rise in inflammation that fails to abate without therapy; an established clinical observation which we explain by suggesting that the loss of wt-CFTR in a CF patient prevents a return to baseline inflammatory signalling. With the caveat that we studied non-polarised cells, wt-CFTR may also need to be ‘active’ because firstly, we find that elevating cAMP suppresses inflammatory markers by about 25% and secondly, that treatment with a specific inhibitor of channel function (CFTR_inh172_) [17], resulted in an increase in NF-κB activity only when wt-CFTR was present (figure 3A). Furthermore, when cells endogenously expressing CFTR were treated with this inhibitor, it not only negated the decline in inflammation found after pharmacological elevation of cAMP but also acted dominantly over our cAMP-elevating cocktail and, separately, in cultured, wt-CFTR expressing primary human nasal cells, induced a near doubling of IL-8 secretion without prior CFTR-stimulation. The repeated dose-dependency of our suppressive findings suggest that wild-type CFTR acts as an expression-level dependent, anti-inflammatory protein. Furthermore, given the reciprocal effects of pharmacological CFTR inhibition (pro-inflammatory) and activation (anti-inflammatory) giving rise to a near 50% difference in levels of a critical hub in inflammation, we concur with Vij et al that that the inflammation suppressive action of wt-CFTR might require an active channel. This may also be relevant to the post-stimulus level of inflammation because of our finding that when cells are stimulated with TNFα, they are much less pro-inflammatory in the presence of wt-CFTR; the latter again acting in a dose-dependent manner suggesting that wt-CFTR suppresses not only basal inflammation but also down regulates TNFα stimulated inflammation (figure 1C). Given that CFTR mRNA is expressed up to 70 times higher in fetal lung than in adult [22], it may be that changes in CFTR levels at the cell membrane can suppress inflammatory pathways. This should not be a surprise as after all, inflammatory pathways found in adult life operate in development to control cell growth and differentiation [23], [24], [25]. Our finding that starting cell number i.e. initial cell density might also be an important signal might also be relevant to the above fetal data where lung cells are rapidly dividing as the lung grows. Importantly, our conclusions are not confounded by differences in transfection efficiency of reporter constructs because they are reproduced to the same degree in HeLa cells stably expressing the NF-κB driven luciferase vector. Crucially, our study independently verifies recent work by Vij and colleagues [15] who showed that re-introduction of wild-type CFTR into a kidney epithelial cell line (HEK293) or, in a disease context, human bronchial epithelial cells expressing the common ΔF508-CFTR (Phe508del) mutation (CFBE41o-) suppresses not only basal NF-κB activity but also had robust inhibitory effects on IL1β-induced chemokine (IL-8) expression [15]. They further demonstrated that exposure to lipopolysaccharide (LPS) evoked a greater immune response in a viable murine CFTR knockout model compared to wild-type animals. Although their experiments also suggest that CFTR functionally suppresses inflammation *in vivo*, the corroborating evidence from cell lines used in their study, when compared to our own data, suggests that the means by which a cell controls the level of wild type CFTR must now be considered as an important contributor, if not a key cause, of inflammation. Thus, we concur that wild type CFTR has an anti-inflammatory function acting through an NF-κB hub that is aberrant in cells expressing mutant CFTR, probably because the most common Phe508del CFTR is >90% degraded, i.e. wt-CFTR is not present as a restraint on inflammation. Although neither study establishes how this suppression of inflammation might occur, each independently verifies that inhibition of CFTR conductance using CFTR_inh172_ mimics the pro-inflammatory effects of the absence of CFTR. Thus, functional CFTR is key to suppressing inflammation. Vij *et al.* propose a model in which active CFTR contained within lipid rafts at the cell membrane associates with TNFα-IL1β-TLR receptors and so tonically represses signalling to the NF-κB pathway. Removal of CFTR relieves this repression and so raises basal activity of the NF-κB pathway. We do not disagree but what exactly constitutes ‘basal’ NF-κB activity is a moot point. Our unpublished data shows that lipofectamine alone when administered to H57 cells for 24 hours induces a significant ∼20% decline in NF-κB activity and this represents a new lower ‘baseline’ compared to lipofectamine-naïve H57 cells. Empty vector did not alter this decline, CFTR made the decline more intense and CFP returned the basal activity towards this original lipofectamine-naïve ‘baseline’. Thus the concept of baseline inflammation requires further study because NF-κB might signal differently in each different ‘baseline’ scenario.

By deliberate intent, our study did not focus on mutant forms of CFTR because the literature is confused and the very existence of inflammation when mutant CFTR is present remains controversial. A number of studies have shown higher expression and secretion of various inflammatory mediators in CF cells compared to wild-type cells, but these are not always homologous CFTR-expressing cells [11], [17], [26], [27], [28], [29], [30], [31], [32], [33]. As a result, there is a great deal of variation between cell lines; different groups have measured a variety of markers in a number of ways and results vary even in the same cell lines, perhaps, as we show here, due to different culture conditions or seeding densities. If inflammation arises from the absence of a basal suppression of inflammation by wt-CFTR, this loss of suppression may operate alongside the positive pro-inflammation function for mutated CFTR reported by many groups. Thus, lung cells expressing high levels of wt-CFTR may be less inflamed than those that express little or no CFTR protein leading us to consider that inflammation decreases in proportion to the amount of wt-CFTR. It has proven difficult to dissect out the pleiotropic effects of CFTR mutants with effects ranging from aberrant turnover, rapid degradation, ER retention and unfolded protein response [34]. For example, under highly-expressing cell conditions, much Phe508del-CFTR fails to traffic properly, and it is expressed in very low levels at the airway surface cell membrane (as opposed to glands). Even this ‘long-established’ idea is controversial because some find mutant CFTR is not mostly degraded [35]. Our data predict that the higher inflammation in CF cells could be a consequence of fewer wt-CFTR molecules at the membrane, as would be predicted to occur with stop mutants such as G542X that occur in a minority of CF patients and the clinical data supports the view that the presence of CFTR mutants such as G551D-CFTR that traffic normally but fail to transport chloride at all, nevertheless reduce lung damage to a degree [36]. This idea is also consistent with reports of the effects of some CFTR ‘correctors’. These drugs increase CFTR expression at the membrane (for example, MPB-07, miglustat and NB-DGJ) and some can reduce the inflammatory response of cells to *Pseudomonas aeruginosa* [37], [38]. With the compounds miglustat and NB-DGJ, this reduction was observed in both CF and non-CF cells. Although both these compounds reduced inflammation, NB-DJG did not restore CFTR pore activity in the CF cells, suggesting that the effects of CFTR on inflammation may be separate from channel opening/closing. These data are also in agreement with the observations of Weber *et al.*, who showed that NF-κB activation and IL-8 secretion were increased in 16HBE14o^−^ cells expressing CFTR antisense construct (compared with those expressing CFTR sense) and in a corresponding experiment in human tracheal cells comparing 9/HTEo^−^/pCep-R cells with 9/HTEo^−^/pCep [33]. In fetal life, CFTR is expressed in the lungs at around 12 weeks gestation, however in the CF fetus Phe508del CFTR expression may be delayed for about 3 weeks [21] and our data predict that NF-κB may be dysregulated for that period. This is in agreement with both Jacquot and colleagues, who find evidence for NF-κB dysfunction in human fetal tracheal grafts in SCID mice [10], and Verhaeghe *et al.* who showed increase expression of various inflammatory markers in human CF fetal lung at 24 weeks gestation [9].

The inhibitor CFTR_inh172_ increased the (lipofectamine, see above) baseline activation of NF-κB and increased IL-8 secretion in 16HBE14o^−^ cells, which is in agreement with Perez *et al.*, who showed that inhibition of CFTR with CFTR_inh172_ increased secretion of IL-8 in 16HBE14o^−^ cells [17]. CFTR_inh172_ is thought to prevent channel opening of CFTR and thus abrogate the chloride current. Recent data, from mutation analysis of critical residues, suggests the inhibitor may act directly on CFTR within the sixth transmembrane helix of the protein, a domain that has an key role in the channel pore formation at arginine 347 [16]; but exactly how the inhibitor acts on CFTR is not fully understood. For example, recently Kelly and colleagues put forward evidence that CFTR_inh172_ had effects on mitochondrial function independent of CFTR [39]. They showed that the inhibitor induced rapid increases in reactive oxygen species (ROS) levels and depolarization of mitochondria in 4 different cell types independent of the presence of CFTR. In HeLa cells with no CFTR present, they found treatment with CFTR_inh172_ increased translocation of NF-κB to the nucleus and a slight increase in IL-8 secretion. However in our hands with the H441 cell line where with no CFTR present we found that there was no significant change in NF-κB activity when treated with the inhibitor, however when CFTR was present there was a significant increase in NF-κB activity, suggesting that the effects of the inhibitor may differ between cell lines, depending on which pathways remain intact (compare figure 3C with 1D). In our hands, treatment with forskolin and IBMX, which normally strongly activates PKA, resulting in opening of the CFTR pore, significantly reduced inflammation in 16HBE14o^−^ cells, indicating that activity of the channel may be required to reduce inflammation. However when CFTR_inh172_ is added, the decrease in inflammation induced by cAMP is completely abrogated. Recent data suggest that CFTR interacts with the TNF receptor TNF-R1 in lipid rafts and modulates IL-8 secretion and gap junction formation [40]. There is also evidence that TNF-R1 is a modulator of CF as there is an association between polymorphisms in the TNF-R1 gene and disease severity [41]. We conclude that CFTR modulates downstream signalling (perhaps from TNF-R1) and suggest that CFTR controls release of inflammatory molecules, resulting in suppression of inflammatory signalling. Since our recent work suggests that CFTR creates a membrane-anchored hub controlling the membrane localisation of at least four different protein kinases, [19], [42], [43], [44], [45], [46], the loss of wild type CFTR at the membrane is predicted to disrupt multiple pathways given that all these protein kinases have been implicated in inflammatory signalling. Further work will have to establish how the dysfunction of this signalling system when mutant CFTR is degraded, leads to multiply disrupted protein kinase cascades, which others have implicated in inflammation [47].

In conclusion, we confirm that expression of exogenous CFTR has a powerful anti-inflammatory effect and that inducers and inhibitors of CFTR function modulate this effect. Different laboratories now agree that the very presence of wild type CFTR suppresses inflammation, making the topic worthy of study. Restoring the mechanism of suppression may help alleviate the commonest cause of lung destruction and morbidity in this disease.

## Materials and Methods

### Ethics Statement

This work was approved by Tayside Committee on Medical Research Ethics Ref 011/91.

### Materials

All tissue culture media and antibiotics were purchased from Sigma Aldrich, Gillingham U.K., fetal calf serum was purchased from Invitrogen, Paisley, U.K. All chemicals were purchased from Sigma Aldrich unless otherwise stated.

### Cell Culture

NCI-H441 cells, a human lung adenocarcinoma cell line of bronchiolar (Clara) cell lineage (obtained from the American Type Culture Collection (ATCC) HTB-174), were grown on plastic tissue culture dishes in RPMI 1640 medium containing 10% fetal bovine serum, penicillin (100 u/ml), streptomycin (100 µg/ml) in a humidified CO_2_ incubator (37°C, 5% CO_2_). H57 cells, a HeLa cell derived cell line which stably over expresses the NF-κB luciferase construct (courtesy of R. Hay, Dundee, UK) [48], and 16HBE14o^−^ cells, a human normal bronchial epithelial cell line provided by D. Gruenert, San Francisco, CA, U.S.A [49] were grown on plastic tissue culture dishes in Eagle's minimum essential medium containing 10% fetal bovine serum, penicillin (100 u/ml), streptomycin (100 µg/ml). All tissue culture media and antibiotics were purchased from Sigma Aldrich, Gillingham U.K., Fetal calf serum was purchased from Invitrogen, Paisley, U.K.

Primary nasal epithelia were grown on plastic tissue culture dishes in BEBM medium (Clonetics) with all Singlequot supplements except EGF and gentamicin, supplemented with 1 mM CaCl_2_, 100 µl of the EGF Singlequot, penicillin (100 U/ml) and streptomycin (100 µg/ml) (Sigma). Ethical permission and written consent were obtained.

### Transient transfection and reporter gene assay

For luciferase assay cells were seeded in 12-well plates. The 4-κB-luciferase reporter plasmid contained four NF-κB binding elements 3′ (TGGGGACTTTCCGC) 5′ upstream of the thymidine kinase minimal promoter region that drove expression of the firefly luciferase reporter gene. H441 and 16HBE14o^−^ cells were transfected with 400 ng of 4-κB-luciferase reporter plasmid using lipofectamine 2000 reagent (Invitrogen, Paisley, UK). H441 cells were transfected with 0–400 ng wt-CFTR and to to rule out a DNA dose effect, transfections were supplemented with empty vector plasmid to keep total DNA constant (for example, the 100 ng transfection of CFTR contained 300 ng of empty vector) during co-transfection with a NF-κB driven luciferase construct (both using lipofectamine as vehicle). H57 cells were treated as H441 cells excluding the luciferase vector. 24 h post transfection, cells were treated with combinations of 10 ng/ml TNFα, to stimulate NF-κB activity, 10 µM CFTR_inh172_ (Merck, Nottingham, U.K.), to inhibit CFTR and 10 µM Forskolin and 10 µM IBMX to stimulate PKA by elevating cAMP. All treatments were for 4 h, after which supernatants were retained for IL-8 ELISAs and cells were lysed in luciferase lysis buffer (10 mM KH_2_PO_4_, 1 mM EDTA, 10 mM MgCL_2_, 50 mM β-glycerophosphate, 5 mM EGTA, 0.5% Igepal, 0.1% Brij35, 1 mM Sodium Orthovanadate and Protease inhibitor cocktail). Reporter gene activity was determined using the luciferase assay system (Promega, Southampton, U.K.).

### IL-8 ELISAs

The concentration of IL-8 in supernatants was determined by ELISA according to the manufacturer's recommendations (Mast Diagnostics, Bootle, U.K.) with appropriate standard curves. Results were normalised for protein content.

### Immunoblotting

Cell lysates were run on 4–12% Novex gels using the MOPS buffer system (Invitrogen UK). Following transfer membranes were blocked in Tris-buffered saline with Tween (TBST) with 5% milk and probed with CFTR Ab-3 (L12B4) antibody (Neomarkers, CA, USA.).

### Reverse Transcription

RNA was extracted using RNeasy Mini Kit (Qiagen, Crawley West Sussex, U.K.). Using 1 µM of oligo dT_15_ primer (Promega, Southampton, U.K.), 2 µl of 5 mM dNTPs, 10 u RNasin® Plus RNase Inhibitor (Promega, Southampton, U.K.) with 4 u of Omniscript reverse transcriptase (Qiagen, Crawley West Sussex), 1 µg of RNA was reverse transcribed by incubating the reaction mixture for 60 min at 37°C.

### PCR

PCR of the cDNA was then carried out using Gotaq green (Promega, Southampton, U.K.) and the CFTR specific primers, exon 9, HCF21: 5′-TTGCTGGATCCACTGGAGCAGG-3′; exons 12–13, HCF22: 5′-GCCATCAGTTTACAGACACAG-3′ which produces a 421bp fragment [50]. 10 µl of each PCR product was run on 1.5% Agarose gels. Negative controls for cDNA synthesis were run without template and also without reverse transcription.

### Real-Time PCR

Real-time PCR(qPCR) was performed using SYBR Green based detection on a Rotor-Gene Q machine (Qiagen) with each 20µl reaction containing approximately 30 ng cDNA, 1×QuantiTect Primer assay (CFTR or B2M) ( Quiagen) and 1×Rotor-Gene SYBR Green PCR master mix (Qiagen). Samples were cycled under the following conditions: 95°C/5min, 50 cycles of 60°C/14 s and 95°C/7 s. each reaction was performed in duplicate, mRNA levels for B2M were used for normalization and fold induction was determined from Ct values using Pfaffl method [51]. PCR efficiency was determined from the slope of a standard curve generated using fivefold dilution series of DNA template. Efficiency values for CFTR 1.01, B2M 1.17, R value of slopes CFTR 0.988 and B2M 0.998.

### Statistics

Statistical significance was assessed relative to control groups using an unpaired Students T-test.
